# Scientists Turn to Journals in English

**DOI:** 10.1100/tsw.2001.57

**Published:** 2001-06-16

**Authors:** A.A. Waheed

**Affiliations:** Department of Protein Biochemistry, Tokyo Metropolitan Institute of Gerontology, 35-2 Sakae-cho, Itabashi-ku, Tokyo 173-0015, Japan

## Abstract

Biomedical publications listed in Medline were analyzed based on publisher’s location [1] and first author’s country of origin [2,3]. In the present analysis I wished to determine the languages of biomedical publications and the publishers’ locations.

Medline was searched with the use of MEDLINE EXPRESS covering every year from 1966 to 1999, selecting the field "language of article" (LA), and combining this with the field "publication year". The publisher's location was obtained by combining the fields LA and "country of publication".

Medline covers articles published in 42 languages, 8 of which are above 1% and account for 95.69% of the total publications (Fig. 1). Publications in 15 languages between 0.1% and 1% include Czech (0.58%), Chinese (0.54%), Danish (0.38%), Portuguese (0.35%), Dutch (0.35%), Swedish (0.34%), Norwegian (0.26%), Hungarian (0.22%), Romanian (0.21%), Serbo-Croatian-Roman (0.19%), Bulgarian (0.16%), Slovak (0.15%), Hebrew (0.12%), Ukrainian (0.12%), and Finnish (0.11%). Publications in 5 languages are 0.01% to 0.1% and lower than 0.01% in 14 languages.

Analysis of publisher's location revealed that, aside from English, German, French, and Spanish, more than 98% of the articles in other languages are published in their respective countries. The major publishers of English language publications are the United States (57.93%), England (20.51%), The Netherlands (5.79%), Germany (5.13%), Switzerland (2.94%), Japan (1.98%), Denmark (1.65%), Canada (1.52%), Australia (1.32%), and India (1.01%); in German, Germany (84.63%) and Switzerland (9.65%); in French, France (77.72%), Belgium (6.99%), Switzerland (6.16%), and Canada (3.66%); and in Spanish, Spain (55.89%), Mexico (15.65%), Argentina (9.35%), Chile (7.95%), Venezuela (2.44%), and the United States (1.85%).

When I analyzed by year, publications in the English language increased from 53.4% in 1966 to 89.67% in 1999 whereas they decreased in other languages: German, 10.9% to 1.95%; Russian, 8.44% to 1.38%; French, 7.71% to 1.90%; Japanese, 3.87% to 1.56%; Italian, 5.23% to 0.49%; Spanish, 1.95% to 1.28%; and Polish, 2.44% to 0.40%. Publications in 34 other languages that contributed 6.06% in 1966 decreased to 1.37% in 1999. Although the decline in other languages is due in part to rapid increase in the number of articles in English, the biased inclusion of English language journals by Medline cannot be ruled out[4], possibly heading for single-language coverage by the year 2015 at this rate. However, the publication policy of biomedical researchers is inclined toward English language journals as analyzed by Egger et al. [5].

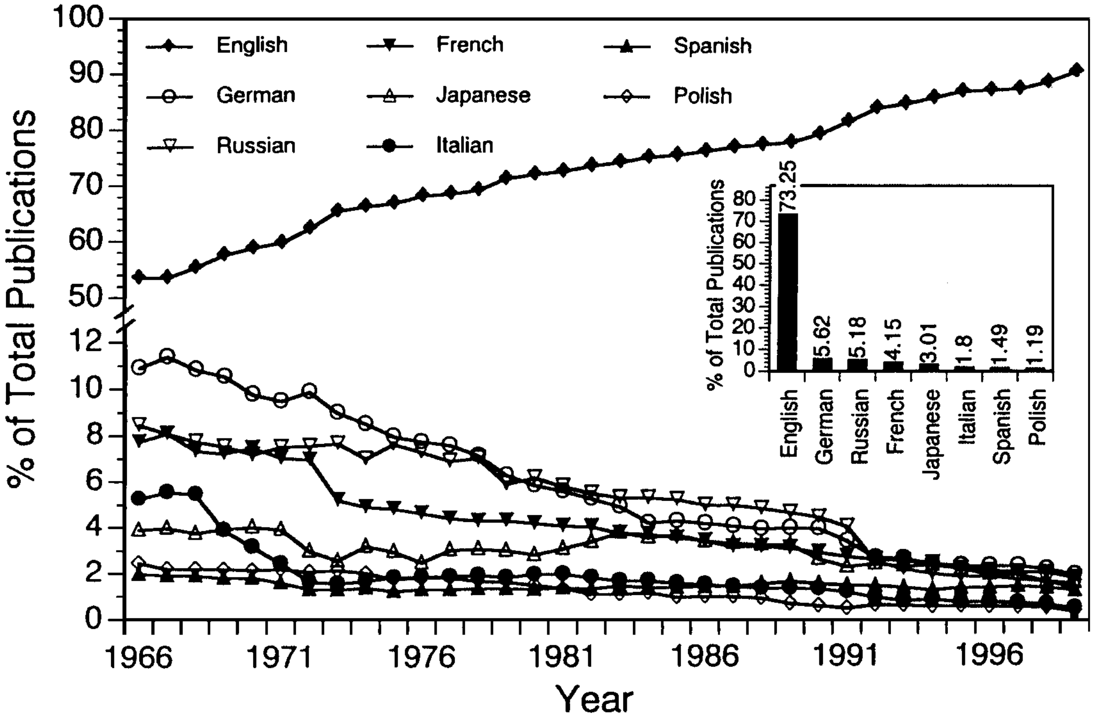

**FIGURE 1.** The percentage of biomedical publications in eight languages that contribute more than 1% of the total (inset) and its year-wise percentage from 1966 to 1999.
